# Tumorigenicity Studies of Induced Pluripotent Stem Cell (iPSC)-Derived Retinal Pigment Epithelium (RPE) for the Treatment of Age-Related Macular Degeneration

**DOI:** 10.1371/journal.pone.0085336

**Published:** 2014-01-14

**Authors:** Hoshimi Kanemura, Masahiro J. Go, Masayuki Shikamura, Naoki Nishishita, Noriko Sakai, Hiroyuki Kamao, Michiko Mandai, Chikako Morinaga, Masayo Takahashi, Shin Kawamata

**Affiliations:** 1 Division of Cell Therapy, Foundation for Biomedical Research and Innovation, Kobe, Japan; 2 Laboratory for Retinal Regeneration, RIKEN Center for Developmental Biology, Kobe, Japan; 3 Department of Ophthalmology, Kawasaki Medical School, Kurashiki, Okayama, Japan; University of Florida, United States of America

## Abstract

Basic studies of human pluripotential stem cells have advanced rapidly and stem cell products are now seeing therapeutic applications. However, questions remain regarding the tumorigenic potential of such cells. Here, we report the tumorigenic potential of induced pluripotent stem cell (iPSC)-derived retinal pigment epithelium (RPE) for the treatment of wet-type, age-related macular degeneration (AMD). First, immunodeficient mouse strains (nude, SCID, NOD-SCID and NOG) were tested for HeLa cells’ tumor-forming capacity by transplanting various cell doses subcutaneously with or without Matrigel. The 50% Tumor Producing Dose (TPD_50_ value) is the minimal dose of transplanted cells that generated tumors in 50% of animals. For HeLa cells, the TPD_50_ was the lowest when cells were embedded in Matrigel and transplanted into NOG mice (TPD_50_ = 10^1.1^, n = 75). The TPD_50_ for undifferentiated iPSCs transplanted subcutaneously to NOG mice in Matrigel was 10^2.12^; (n = 30). Based on these experiments, 1×10^6^ iPSC-derived RPE were transplanted subcutaneously with Matrigel, and no tumor was found during 15 months of monitoring (n = 65). Next, to model clinical application, we assessed the tumor-forming potential of HeLa cells and iPSC 201B7 cells following subretinal transplantation of nude rats. The TPD_50_ for iPSCs was 10^4.73^ (n = 20) and for HeLa cells 10^1.32^ (n = 37) respectively. Next, the tumorigenicity of iPSC-derived RPE was tested in the subretinal space of nude rats by transplanting 0.8–1.5×10^4^ iPSC-derived RPE in a collagen-lined (1 mm×1 mm) sheet. No tumor was found with iPSC-derived RPE sheets during 6–12 months of monitoring (n = 26). Considering the number of rodents used, the monitoring period, the sensitivity of detecting tumors via subcutaneous and subretinal administration routes and the incidence of tumor formation from the iPSC-derived RPE, we conclude that the tumorigenic potential of the iPSC-derived RPE was negligible.

## Introduction

Clinical cell therapy trials were recently initiated for treatment of Stargardt’s disease and the dry type of age-related macular degeneration (dry AMD). The trials have used human embryonic stem cell (hESC)-derived retinal pigment epithelium (RPE) [1]–[4]. In addition, several groups are planning clinical trials with autologous human induced pluripotent stem cell (hiPSC)-derived RPE for the wet type of AMD. Thus, cell therapy using human pluripotent stem cells (hPSCs) has reached clinical application. However, in contrast to tissue stem cells that have a limited proliferation potential, tumor formation from residual undifferentiated or incompletely differentiated hPSCs in hPSC-derived cell products is an issue that must be carefully analyzed. This issue is particularly important when transplanting autologous hiPSC-derived cells.

We recently reported a highly sensitive residual hiPSC detection method based on qRT-PCR using primers for the *LIN28A* transcript [5] in hiPSC-derived RPE. This method enables us to detect residual hiPSCs down to 0.002% of differentiated RPE cells. As we plan to transplant 4–8×10^4^ hiPSC-derived RPE cells into the subretinal space of patients, this method is sensitive enough to detect a few residual hiPSCs, if any, in a clinical setting.

The tumorigenic potential of hiPSC-derived RPE cells is attributable to contamination by undifferentiated hiPSCs, intermediate products having proliferation potentials and/or tumorigenic transformed cells. Contamination by these cells should be assessed by nonclinical testing using suitable animal models [6], [7]. However, there is no internationally recognized guideline for tumorigenicity testing in cell therapy products. The most relevant guideline is the WHO TRS 878, “Recommendation for the evaluation of animal cell cultures as substrates for the manufacture of cell banks” [8], [9]. The guideline recommends transplanting 1×10^7^ test cells subcutaneously to 10 nude mice and monitoring tumor formation for more than 16 weeks. Transplantation of the same dose of a well-known tumorigenic cell line such as HeLa in parallel is suggested as a tumor-forming positive control. The WHO guideline covers animal cell substrates for the production of biological medicinal products and specifically excludes viable animal cells that are intended for therapeutic transplantation into patients. To examine the tumorigenicity of hiPSC-derived cells intended for administration to patients, several teratoma-forming tests exploring dose and administration route were studied using immuno-deficient mice [6],[10]. However, discussions how we can interpret and extrapolate the results of tumorigenicity testing with immuno-deficient or immuno-suppressant animals to human patients continue [6], [7]. Recently a commentary report from FDA/CBER pointed out the issues to be considered for cell-based products and associated challenges for preclinical animal study [11]. The report stated that although the nature of cells used for cellular therapy is diverse, tumorigenic test results from the administration of cells through nonclinical routes would not be considered relevant as it would not track the behavior of transplanted cells in a micro-environment. When tumorigenicity testing of ESC-derived cellular products is undertaken, the study design should include groups of animals that have received undifferentiated ESCs, serial dilutions of undifferentiated ESCs combined with ESC-derived final products and the final intended clinical products. This approach would thereby address the tumor-forming potential of these cell groups in animal models.

Tumorigenicity testing via the clinical route of administration could recapitulate the fate of transplanted cells in a microenvironment of host tissue and could be fairly extrapolated to human application. However, elaborate surgical intervention requires skills that greatly influence the outcome of transplantation. For example, it is difficult to determine whether the cells were transplanted into the right location or organ in small rodents. These concerns can be overcome by conducting a subcutaneous tumorigenicity test in addition to testing via the clinical route.

In this report, we conducted 2 types of *in vivo* tumorigenicity tests by transplanting hiPSC-derived RPE cells into subcutaneous and sub-retinal spaces in immuno-deficient animals. The results and limits of these tests are discussed.

## Results

### Tumorigenicity Tests with Several Types of Immuno-deficient Mice

The tumor-forming potential of human iPSC-derived cell products should be examined using a suitable animal transplantation model. One should take into account the number of cells to be transplanted, the method of transplantation, the microenvironment of the transplantation site, the monitoring period and the status of the immune-deficient animals.

**Figure 1:**
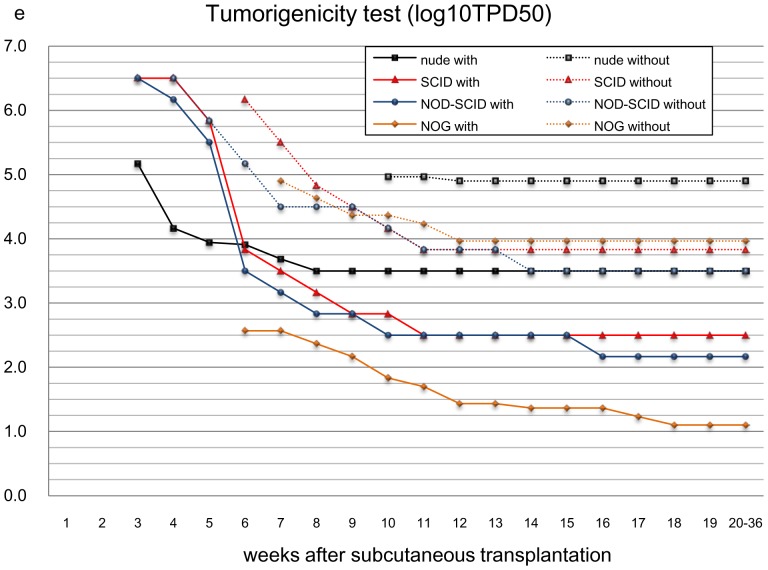
Tumorigenicity testing (TPD_50_ log_10_) by subcutaneous transplantation of HeLa cells. Log_10_TPD_50_ values (minimal cell doses for 50% of animals to form a tumor) for HeLa cells when transplanted subcutaneously in various immuno-deficient mouse strains (nude, SCID, NOD-SCID, NOG) with or without Matrigel as indicated. Abscissa, weeks after transplantation. Ordinate, Log_10_ TPD_50_ values, logarithmic scale.

First, we checked the tumor-forming potential of several immune-deficient animals by subcutaneously transplanting HeLa cells over a wide range of doses (1 to 1×10^6^ in 10-fold increments) with or without Matrigel (BD) and observed tumor formation every day for up to 36 weeks on a daily basis. Matrigel is known to enhance the tumor-forming potential of transplanted cells [16]. Recipient animals included immune-deficient nude, SCID [10], NOD-SCID [17], and NOG [18] mice. The minimal dose of transplanted cells that generated tumors in 50% of the transplanted animal (TPD_50_) was calculated statistically to evaluate the sensitivities of tumor formation in each animal model [19]. We found the NOG mouse was most susceptible to tumors. That is, when transplanted subcutaneously with Matrigel, tumors were generated by the lowest number of HeLa cells. The TPD_50_ for HeLa was 10^1.1^ (n = 75), in agreement with a previous report [20], (Figure 1, Table 1). It is interesting to note that among the conditions tested, the highest number of HeLa cells was required to form tumors in nude mice without Matrigel. TPD_50_ for nude mice without Matrigel was 10^4.9^ (n = 120), which is also in agreement with a previous report [19] (Figure 1, Table 1). Therefore, we selected NOG mice and Matrigel for embedding the test cells for further assays as it provided sensitive tumor detection using small numbers of transplanted cells. The tumor-formation potential of iPSCs was assessed by subcutaneously transplanting several doses of the iPSC cell line 201B7 with Matrigel into NOG mice. The TPD_50_ for iPSC was 10^2.12^ (n = 30) over 12 months’ monitoring (Table 2). The TPD_50_ value for iPSCs in subcutaneous transplantation provided a reference cell number for the contamination of iPSCs in iPSC-derived RPE cells.

**Table 1:** Incidence of tumor formation after transplanting HeLa cells in various immunodeficient mice.

strain	use of Matrigel	min.dose fortumor formation	weeks to observeTumor (first to last)	number of mice	Log10TPD50
nude	with	1×10^4^ cells	3 to 8	120	3.5
nude	w/o	1×10^4^ cells	4 to 12	120	4.9
SCID	with	1×10^3^ cells	3 to 11	24	2.5
SCID	w/o	1×10^3^ cells	3 to 11	24	3.83
NOD-SCID	with	1×10^2^ cells	3 to 16	24	2.17
NOD-SCID	w/o	1×10^3^ cells	3 to 14	24	3.5
NOG	with	1×10^1^ cells	5 to 18	75	1.1
NOG	w/o	1×10^4^ cells	3 to 13	105	3.97

Log_10_TPD_50_ values for HeLa cells transplanted subcutaneously into various immunodeficient mouse strains with or without Matrigel. Tumor-forming potentials of HeLa cells in nude mice without Matrigel and in NOG mice with Matrigel are highlighted in gray.

**Table 2:** Tumorigenicity testing by subcutaneous transplantation of hiPSC-derived RPE into NOG mice.

hiPSC cell line	cell form	min.dose fortumor formation	weeks to observeTumor (first to last)	numberof mice	Log10TPD50
201B7	Cell suspension in Matrigel	1×10^1^ cells	5–40	30	2.12
**RPE cell line**	**cell form**	**number of cells** **transplanted**	**monitor** **period**	**number** **of mice**	**tumor** **formation**
59-G3(1)	RPE cell suspension in Matrigel	1×10^6^ cells	26–84 weeks	9	none
K21-G18	RPE cell suspension in Matrigel	1×10^6^ cells	26–74 weeks	8	none
101-G25	RPE cell suspension in Matrigel	1×10^6^ cells	23–70 weeks	10	none
59-G3(1)	RPE cell sheet in Matrigel	1×10^6^ cells	28–85 weeks	5	none
K21-G18	RPE cell sheet in Matrigel	1×10^6^ cells	13–79 weeks	5	none
101-G25	RPE cell sheet in Matrigel	1×10^6^ cells	23–79 weeks	5	none
primary RPE	Cell suspension in Matrigel	1×10^6^ cells	52 weeks	3	none
primary RPE	Cell suspension w/o Matrigel	1×10^6^ cells	52 weeks	2	none
59-G3(2)	RPE cell sheet in Matrigel	1×10^6^ cells	26–50 weeks	3	none
RNT10	RPE cell sheet in Matrigel	1×10^6^ cells	26–46 weeks	3	none
RNT9	RPE cell sheet in Matrigel	1×10^6^ cells	26–38 weeks	3	none
101-EV3	RPE cell suspension in Matrigel	1×10^6^ cells	39 weeks	5	none
K11-EV9	RPE cell suspension in Matrigel	1×10^6^ cells	39 weeks	3	none
K21-EV15	RPE cell suspension w/o Matrigel	1×10^6^ cells	39 weeks	4	none
K11-EV9	RPE cell suspension w/o Matrigel	1×10^6^ cells	39 weeks	2	none

Log_10_TPD_50_ value for hiPSC 201B7 determined by subcutaneously transplanting cells in Matrigel into NOG was calculated by the Trimmed Spearman-Karber method (upper panel). Tumor formation from 1×10^6^ hiPSC-derived RPE cells prior to making RPE sheets (cell suspension) or after making RPE sheets (cell sheet) transplanted subcutaneously in various conditions into NOG mice. Animals were monitored for 13–85 weeks (lower panel).

### Characterization of Established hiPSCs and hiPSC-derived RPE

hiPSC lines 59-G3, 101-EV3, K11-EV9, K21-EV15 K21-G18, 101-G25, RNT9-2-8, and RNT10–24 were established from dermal fibroblasts of 6 patients (59, K11, K21, 101, RNT9, RNT10) with retinitis pigmentosa. Quality control tests for established iPSCs were as follows. (1) Cells form colonies and must show human ESC-like morphology by microscopic observation. (2) Cells must express SSEA-4, TRA-1-60, POU5F1 (OCT3/4) and NANOG proteins as determined by immunostaining. (3) Cells must not express EBNA plasmid fragment by PCR or qRT-PCR. (4). Cells must possess a normal karyotype by the G-band method.

**Figure 2:**
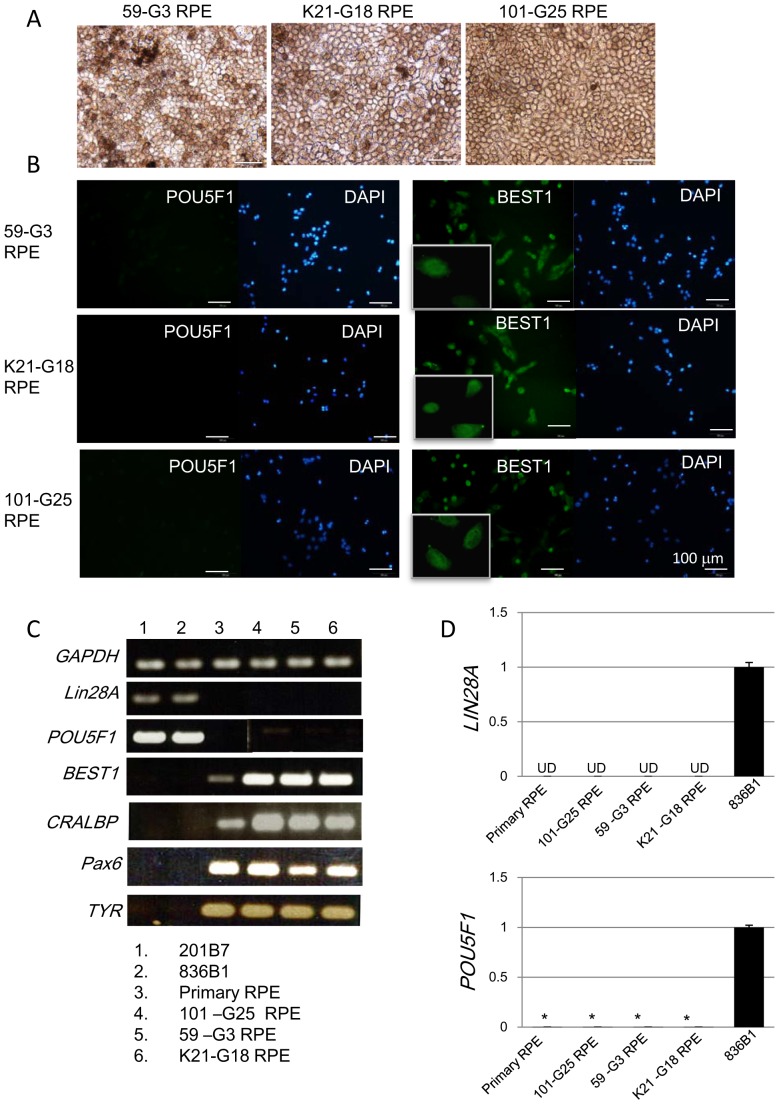
Characterization of hiPSC-derived RPE. A: Phase contrast images of hiPSC-derived RPE cell lines 59-G3 RPE, K21-G18 RPE and 101–G25 RPE. B: Expression of pluripotency-related molecules POU5F1 (OCT3/4, upper panels) and RPE-related molecules BEST1 (lower panels) in lines 59-G3 RPE, K21-G18 RPE and 101–G25 RPE as detected by immunostaining with specific antibodies. Nuclei were stained with DAPI. Magnified photos of BEST1 staining are appended in left lower corners. C: Gene expression profiles of hiPSC-derived RPE cell lines 59-G3-RPE, K21-G18 RPE, 101-G25 RPE. Expression of pluripotent stem cell-related gene markers *LIN28A* and *POU5F1*, or RPE-related makers *BEST1* (bestrophin), *CRALBP, PAX6* and *TYR* (tyrosinase) in hiPSC cell lines 201B7 and 836B1, primary RPE (hRPE-1) and hiPSC-derived RPE cell lines 59-G3 RPE, K21-G18 RPE, 101-G25 RPE as determined by RT-PCR (left panel). *GAPDH* was used as an internal control. 50 ng RNA was used for one RT reaction. Gene expression of *LIN28A* (D) or *POU5F1* (E) in hiPSC-derived RPE cell lines 59-G3 RPE, K21-G18 RPE, 101-G25 RPE, hiPSC 836B1 and primary RPE was quantified by qRT-PCR. UD: undetectable level (D). *, *P*<0.005 (E). *P* values for primary RPE, 101-G25 RPE, 59-G3 RPE, or K21-G18 RPE versus 836B1 are 0.000153, 0.000177, 0.000432 or 0.000489, respectively.

Retinal differentiation was subsequently initiated. The resulting RPE cell lines were established as follows. 59-G3 RPE was derived from hiPSC clone 59-G3; 101-EV3 RPE was from 101-EV3; K11-EV9 RPE was from K11-EV9; K21-EV15 RPE was from K21-EV15; K21-G18 RPE was from K21-G18; 101-G25 RPE and RNT9 RPE were from RNT9-2-8; and, RNT10 RPE was from RNT10-24. The protocol for RPE differentiation from hiPSC was shown in our recent report [21]. It requires 3 months for RPE differentiation and another 2 months to prepare the RPE sheet. The following quality control tests for the hiPSC-derived RPE cell lines were conducted. (1) The EBNA plasmid fragment was not detectable by PCR. (2) The cells showed the characteristic morphology and pigmentation of RPE with a single or double layer cell structure. (3) BEST1 and PAX6 molecules were detected by immunohistochemistry in over 95% of final hiPSC-derived RPE cells. (4) RPE-specific markers *RPE65, CRALBP, MERTK* and *BEST1* were confirmed by RT-PCR. (5) *LIN28A* was not detected by qRT-PCR. (6) Migration of non-RPE cells into the collagen layer lining the hiPSC-derived RPE cell sheet shall be below 0.1% of the total RPE cells. (7) The RPE cell sheet shall consist of over 70% viable cells with a density of over 4500 cells/mm^2^. Items (6) and (7) were quality control tests for the RPE cell sheet. All the cell culture processes including establishment of hiPSCs from a patient’s fibroblasts and differentiation to RPE were conducted in a GMP-grade cell processing facility. The morphology and immunostaining of hiPSC-derived RPE cell lines 59-G3 RPE, K21-G18 RPE and 101-G25 RPE are shown in Figure 2A, B. The other hiPSC-derived RPE cell lines showed the same phenotype. The gene expression patterns of these cell lines are shown in Figure 2C. Primary RPE was used as a reference. It is notable that neither *LIN28A* nor *POU5F1 (OCT3/4)* was detected above background levels in hiPSC-derived RPE cells^5^. This finding serves as a useful criterion to eliminate immature hiPSCs in hiPSC-derived RPE (Figure 2D, 2E).

### Tumorigenicity Testing of hiPSC-derived RPE (Subcutaneous Transplantation)

To assess the tumorigenic potential of hiPSC-derived RPE cells with high sensitivity, subcutaneous transplantation of a large number of RPE cells would be ideal. However, the maximal number of RPE cells available for transplantation was limited by the culture capacity of the cell processing facility. We hypothesized that transplanting 1×10^6^ hiPSC-derived RPE cells was an acceptable cell number to address the tumorigenic potential of the final cell product when embedded in Matrigel in NOG mice. This hypothesis was based on the facts that we expect to transplant 4–8×10^4^ hiPSC-derived RPE in a clinical setting and as few as 10 undifferentiated hiPSCs or HeLa cells embedded in Matrigel could generate tumors in NOG mice (Table 1, Table 2).

**Figure 3:**
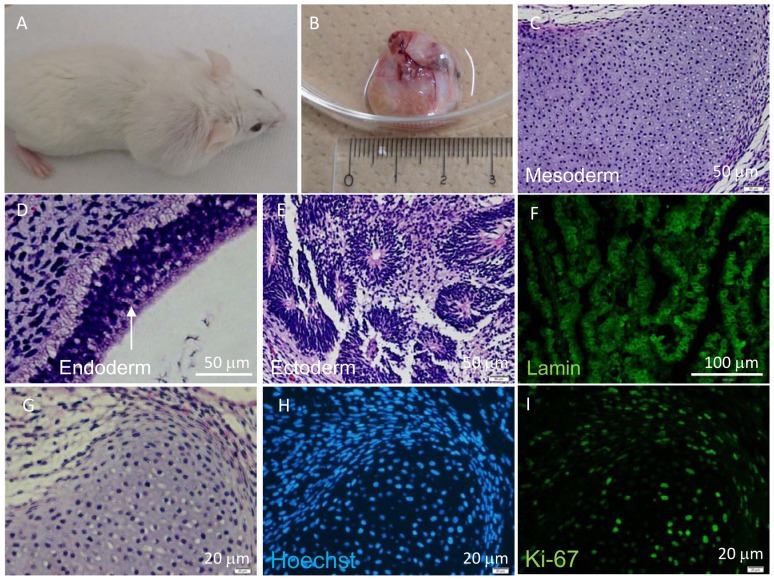
Histological analyses of hiPSCs subcutaneously transplanted into NOG mice. Tumor (teratoma) in NOG mouse was detected 5 weeks after transplanting 1.0×10^4^ hiPSCs embedded with Matrigel (A, B). HE staining of sectioned hiPSC-derived teratoma consisted of three germ layers: cartilage-like tissue (mesoderm) (C), intestinal epithelium-like tissue (endoderm) (D) and neural rosette-like tissue (ectoderm) (E). Anti-Lamin A antibody (F) staining of rosette-like tissue. HE (G), Hoechst 33258 (H) and anti-Ki-67 antibody (I) staining of cartilage-like tissue.

Thus, we subcutaneously transplanted 1×10^6^ hiPSC-derived RPE cells embedded in Matrigel into NOG mice [total n = 42; 59-G3 RPE (n = 14), K21-G18 RPE (n = 13), 101-G25 RPE (n = 15)]. Tumor formation was monitored for more than 70 weeks. Teratoma derived from subcutaneously transplanted iPSCs was analyzed as a positive control for tumor formation event (Figure 3A–3E). The proliferative status of living cells was assessed by HE, Hoechst 33258 and anti-Ki67 antibody staining (Figure 3G–3I). iPSC-derived neural rosette-like human cells were stained by anti-Lamin A antibody to check the specificity of anti-Lamin A antibody for human cells (Figure 3F). We assumed that anti-human Lamin A antibody could stain a wide range of human cell types and was not limited to human RPE. Transplantation of 1×10^6^ primary RPE cells embedded in Matrigel (n = 3) was used as a transplantation control. No tumor formation was observed from transplanted 1×10^6^ hiPSC-derived RPE of several origins in various administration forms. All of the subcutaneous tumorigenicity tests conducted for hiPSC-derived RPE under various conditions using NOG mice are shown in Table 2.

**Figure 4:**
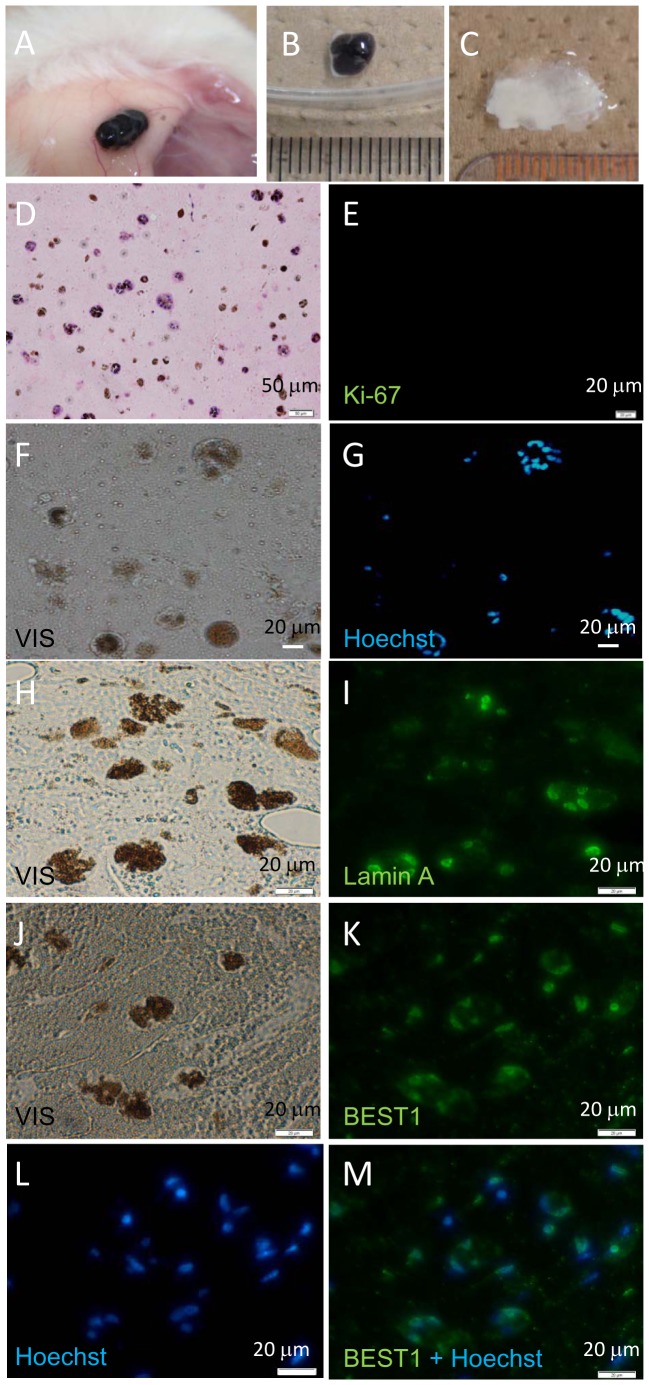
Histological analysis of hiPSC-derived RPE transplanted subcutaneously into NOG mice. NOG mice were examined six months after transplantation of 1.0×10^6^ hiPSC-derived RPE cells in Matrigel into subcutaneous tissue. No tumor was detected visually. Site of transplant (A), excised transplant (B), and excised Matrigel only transplant (Matrigel without RPE cells C). Transplants were sectioned and stained with HE (D) and anti-Ki67 antibody (E). Photomicrograph of unstained serial section (F), and section stained with Hoechst 33258 (G). Photomicrograph of unstained serial section (H) or stained with anti-Lamin A antibody (I). Photomicrograph of unstained serial section (J) or stained with anti-BEST1 antibody (K) and Hoechst 33258 (L) and merged (M). Ki-67 positive cells were not observed.

**Figure 5:**
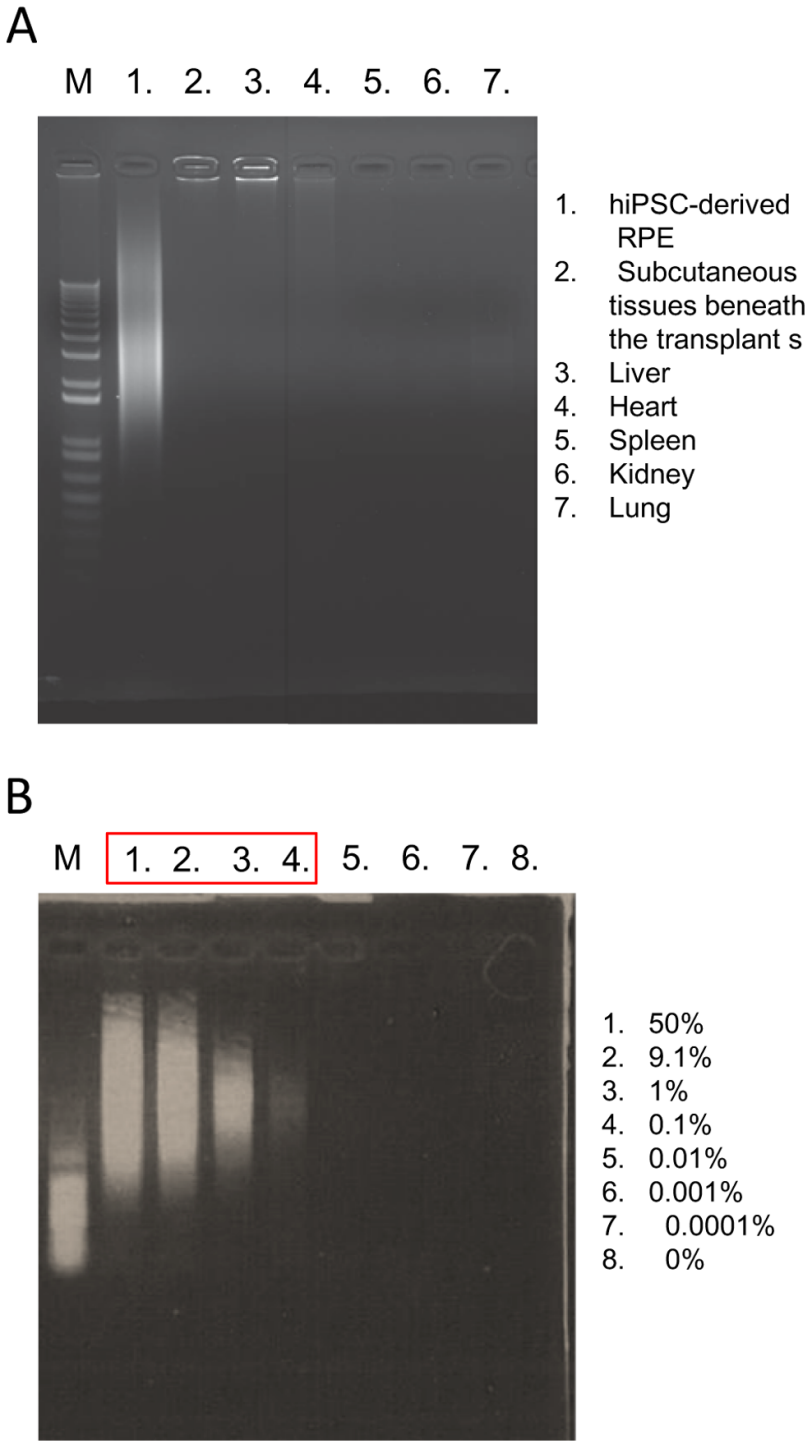
Detection of human cells in host mouse tissue by *Alu* PCR. DNA from hiPSC-derived RPEs (positive control, Lane 1), NOG mouse subcutaneous tissue just beneath the transplants (2), mouse liver (3), mouse heart (4), mouse spleen (5), mouse kidney (6) and mouse lung (7) were used as PCR templates. M: 1 kb marker (A). *Alu* PCR detects ≥0.1% human cells included in mouse cells determined by visual assessment of PCR products generated from various ratios of human: mouse DNA template mixtures. Percentage of human DNA in DNA mixture is shown in a respective lane number (1–8) (B). M: 1 kb marker.

All subcutaneous transplants consisting of RPE cells embedded in Matrigel were excised and subjected to histological examination. The size of transplants in subcutaneous tissue (Figure 4A, 4B) was similar to that of Matrigel without RPE (Figure 4C). Histological and immunohistological study showed that Lamin A-, BEST1- and Hoechst-positively staining RPE cells were present in all the Matrigel transplants (Figure 4F–4M). None of the cells transplanted in Matrigel stained with anti-Ki67 antibody, suggesting the absence of active proliferation in these transplanted cells (Figure 4D, 4E). Human cells derived from transplanted iPSC-derived RPE could be detected by *Alu* PCR at a level of ≥0.1% in mouse cells. However, we could not detect human cells in subcutaneous mouse tissue just beneath the transplants, in liver, spleen, kidney or lung by this method (Figure 5A, 5B).

### Tumorigenicity Test of hiPSC-derived RPE (Subretinal Transplantation)

**Figure 6:**
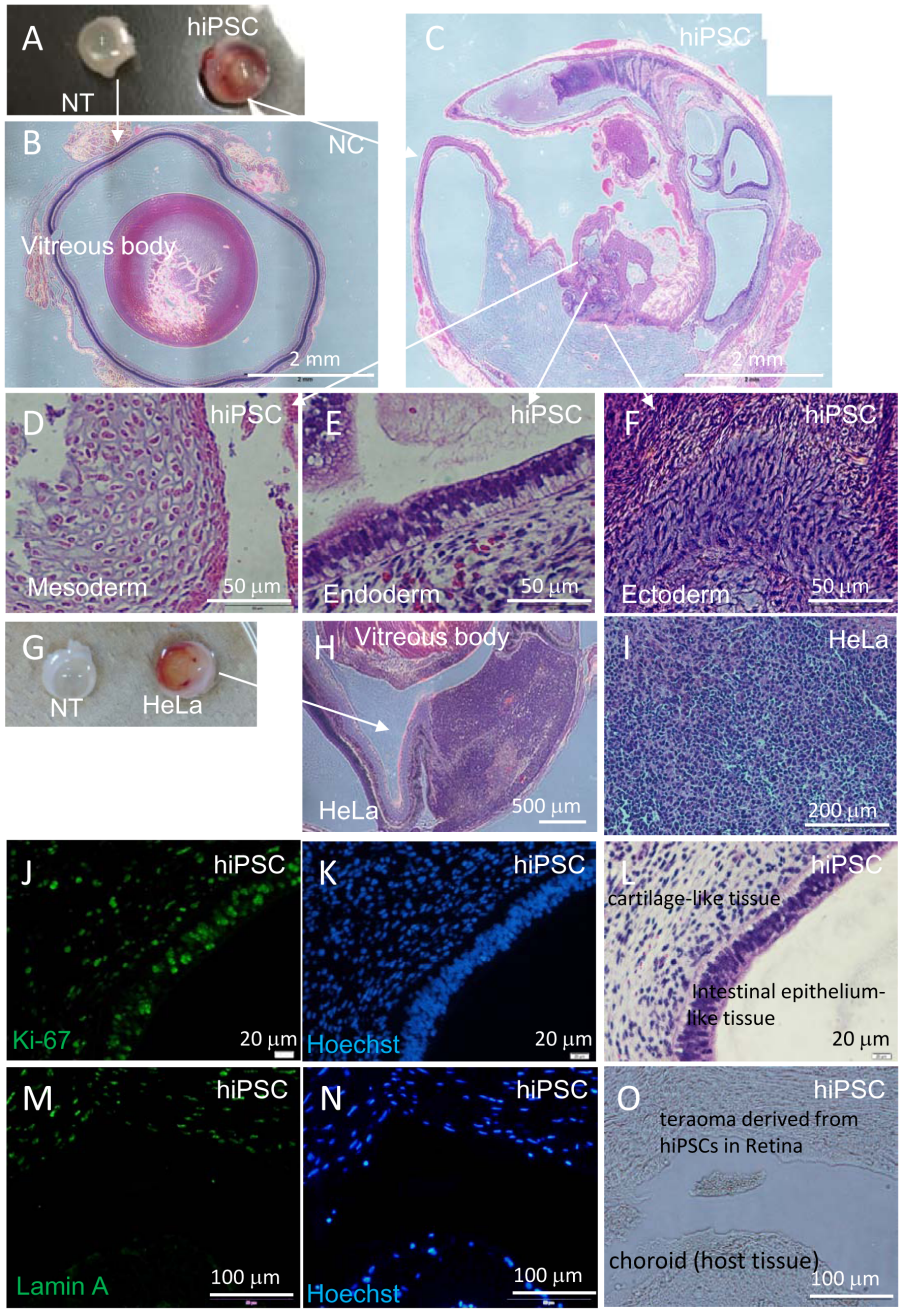
Histological analyses of hiPSCs or HeLa cells transplanted into the subretinal space of nude rats. Eye balls were excised from a nude rat 7 weeks after subretinal transplantation of hiPSC. Non-transplanted right eye ball (NT) and left eye ball transplanted with 1×10^4^ hiPSCs (hiPSC) (A). HE staining of cross section of NT eye ball (B) and hiPSC-transplanted eye ball (C). HE staining of hiPSC-derived teratoma with three germ layers: cartilage-like tissue (mesoderm, D), intestinal epithelium-like tissue (endoderm, E) and neuron-like tissue (ectoderm, F) in hiPSC-transplanted eye ball. (G – O) Eye balls were excised from a nude rat 5 weeks after subretinal transplantation of HeLa cells. Non-transplanted right eye ball (NT) and left eye ball transplanted with 1×10^5^ HeLa cells (HeLa) (G). HE staining of cross section of HeLa cell-transplanted eye ball (H) and HeLa-derived tumor tissue (I). Anti-Ki-67-antibody (J), Hoechst 33258 (K) and HE staining (L) of serial sections of hiPSC-derived teratoma. Anti-Lamin A antibody (M), Hoechst 33258 (N) staining and microscopic image (O) of serial cross sections containing a boundary of hiPSC-derived teratoma and host rat tissue. Anti-Lamin A antibody specifically recognizes human cells in rat tissue.

Next, we conducted tumorigenicity tests by transplanting test cells into the subretinal space, a procedure that is technically demanding. We chose large albino nude rats to facilitate transplantation and minimize variability of test results. This choice also permitted us to transplant larger doses of human hiPSC-derived RPE cells to the subretinal spaces. First, we assessed the tumor-formation potential of HeLa and iPSC 201B7 via subretinal transplantation. The TPD_50_ for HeLa was 10^1.32^ (n = 37) and 10^4.73^ for iPSC (n = 20) (Table 3). Teratomas derived from subretinally transplanted iPSC or tumors derived from transplanted HeLa cells were analyzed as positive controls for tumor formation event in the subretinal space (Figure 6A–6I). The proliferative status of living cells was assessed by HE, Hoechst 33258 and anti-Ki-67 antibody staining (Figure 6J–6L). iPSC-derived human cells were stained by anti-Lamin A antibody to check the specificity of anti-human Lamin A antibody to human cells (Figure 6M–6O).

**Table 3:** Tumorigenicity testing by subretinal transplantation of hiPSC-derived RPE in nude rats.

hiPSC cell line	cell form	min.dose fortumor formation	weeks to observeTumor (first to last)	numberof rats	Log10TPD50
HeLa	Cell suspension w/o Matrigel	1×10^1^ cells	5–33	37	1.32
hiPSC 201B7	Cell suspension w/o Matrigel	1×10^4^ cells	7–33	20	4.73
**RPE cell line**	**cell form**	**number of cells** **transplanted**	**monitor** **period**	**number** **of rats**	**tumor** **formation**
59-G3 (1)	RPE cell sheet w/o Matrigel	0.8–1.5×10^4^ cells	9–82 weeks	4	none
K21-G18	RPE cell sheet w/o Matrigel	0.8–1.5×10^4^ cells	9–82 weeks	4	none
101-G25	RPE cell sheet w/o Matrigel	0.8–1.5×10^4^ cells	9–82 weeks	3	none
59-G3 (2)	RPE cell sheet w/o Matrigel	0.8–1.5×10^4^ cells	8–50 weeks	5	none
RNT10	RPE cell sheet w/o Matrigel	0.8–1.5×10^4^ cells	26–47 weeks	5	none
RNT9	RPE cell sheet w/o Matrigel	0.8–1.5×10^4^ cells	12–38 weeks	5	none

Log_10_TPD_50_ values for HeLa cells or for hiPSC 201B7 cells following subretinal transplantation to nude rats (upper panel). Subretinal tumorigenicity tests conducted using nude rats under various conditions (lower panel).

Next, we conducted tumorigenicity tests of iPSC-derived RPE by transplanting 1 mm^2^-sized (1 mm×1 mm) iPSC-derived RPE sheets consisting of 0.8–1.5×10^4^ RPE cells into the subretinal space of nude rats (n = 26). The RPE cell number was assessed by cell density and sheet size transplanted. Considering the relative sizes of humans and rats, we estimated that transplanting 0.8–1.5×10^4^ RPE cells into the subretinal space of nude rats would provide the information required to determine the incidence of tumor formation in humans, as we expect to transplant 4–8×10^4 ^RPE cells in a clinical setting. Thus, we transplanted 5 different hiPSC-derived RPE cell sheets from 5 different patients to minimize individual variations. hiPSC-derived RPE sheets [59-G3 RPE (n = 9), K21-G18 RPE (n = 4), 101-G25 RPE (n = 3), RNT9 RPE (n = 5), RNT10 RPE (n = 5)] were prepared and transplanted under various conditions. Transplanted nude rats were monitored for tumor formation and physical condition daily for 8 to 82 weeks.

**Figure 7:**
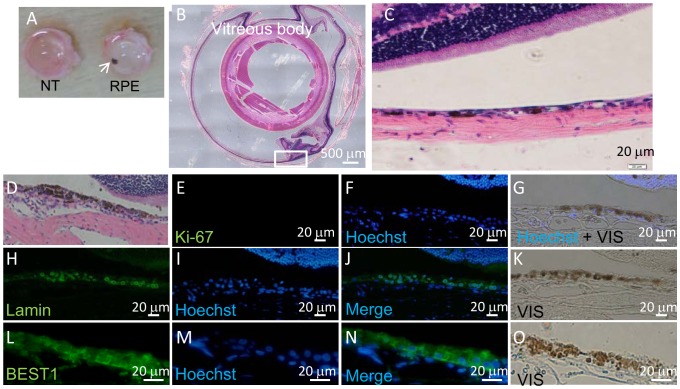
Histological analysis of hiPSC-derived RPE sheets transplanted into the subretinal space of nude rats. (A) Eye balls of nude rat 9 months after subretinal transplantation of 0.8–1.4×10^4^ hiPSC-derived RPE (in a 1 mm×1 mm cell sheet). Left eye ball transplanted with hiPSC-derived RPE (RPE) and non-transplanted right eye ball (NT). (B) HE staining of cross section of left eye ball following transplantation of hiPSC-derived RPE. (C) HE staining of section of eye ball following transplantation of hiPSC-derived RPE, high magnification. HE- (D), anti-Ki67 antibody- (E), and Hoechst 33258-staining (F) and merged (G) images of serial sections of nude rat retina after transplantation of hiPSC-derived RPE. Anti-Lamin A antibody (H), Hoechst 33258 staining (I), merged (J) and micrographic image (K) of serial sections of nude rat retina after transplantation of hiPSC-derived RPE. Anti-BEST1 antibody (L), Hoechst 33258 (M), merged (N) staining and micrographic image (O) of serial sections of nude rat retina following transplantation of hiPSC-derived RPE.

No tumor was found during the period of observation (Table 3). All transplanted eye balls were excised and subjected to histological examination. The location of transplanted RPE sheet was shown by the brown color of the RPE sheet in albino nude rats (Figure 7A–7B). Histological and immunohistological studies showed that Lamin A- and Hoechst-positively staining or BEST1- and Hoechst-positively staining transplanted RPE cells were present in the subretinal space (Figure 7H–7O). Although we used serial sections for this staining, we believe more than half of Lamin A positive-human cells were stained with Hoechst, suggesting that these cells were live human transplanted cells at the end of the experiment (Figure 7H–7K). However, none of the cells in the sub-retinal space was stained with anti-Ki-67 antibody, suggesting that there was no ongoing proliferation in transplanted RPE cells (Figure 7D–7G). Histological analysis of serial sections showed that the shape of hiPSC-derived RPE sheet was maintained after transplantation and no evidence of tissue invasion or destruction of the vicinity of retinal structure was observed (Figure 7C, 7D).

## Discussion

Here, we presented the results of nonclinical tests assessing the tumorigenic potential of hiPSC-derived RPE sheets. These studies represent a portion of the nonclinical testing of our scheduled clinical study for the use of autologous hiPSC-derived RPE sheets for the treatment of wet type AMD. The clinical study is scheduled to commence in 2014. The hiPSC-derived RPE cells used in this study were prepared in a GMP-grade cell processing facility using the same procedures that will be used for patient treatment.

Two types of tumorigenicity tests are summarized in this report. The first was a subcutaneous tumorigenicity test in NOG mice using Matrigel and the second was a subretinal tumorigenicity test in nude rats. It is intriguing to compare the objectives and the validity of the 2 tests in regard to hiPSC-derived RPE cell transplantation. Rationales for conducting subcutaneous tumorigenicity test are as follows:

1. Large numbers of test cells can be transplanted without difficulty, and bias-inducing variations in technical skills can be neglected. Moreover, the tumors are easy to detect. Therefore, statistical and endpoint analysis (TPD_50_ assessment) can be conducted in a timely and accurate manner.
2. It is possible to conduct comparison studies of the tumor-forming potential of different cellular products under the same transplantation conditions.
3. Above all, this test could serve as a substitute for *in vitro* soft agar assays of PSCs. PSCs cannot survive in soft agar, so that mode of testing is not feasible [5]. In contrast, PSCs or PSC-derived cells can survive long-term (more than 12 months) in Matrigel when subcutaneously transplanted in NOG mice. We can detect tumors derived from as few as 10 iPSCs or HeLa cells in this system (Table 1, Table 2). Thus, it provides a highly sensitive tumorigenic test for detecting both residual hiPSCs and tumorigenic transformed cells in hiPSC-derived cell products.

In this context, subcutaneous transplantation testing can be considered a quality control test of final cell products to ensure the absence of tumorigenic cells rather than characterizing the tumorigenic potential of the final cell products at a clinical transplantation site.

Next, we conducted tumorigenicity tests of hiPSC-derived RPE via clinical administration route. Under physiological conditions, the RPE is a monolayer that secretes various cytokines to maintain its structure in the retina. Diniz et al [3] reported that RPE transplanted in sheets retain better survival than when transplanted in suspension. For clinical application, we plan to transplant hiPSC-derived RPE in a sheet form and our preclinical testing was designed accordingly. Although we do not have RPE cell survival data in suspension form, it would be logical to presume that the transplantation of RPE in a sheet form exerts physiological function more effectively than in suspension, which may also facilitate the adaption of transplanted cells to the subretinal tissues.

With regard to the tests’ ability to detect immature (undifferentiated) hiPSCs in the subretinal space as a growing tumor, we demonstrated the trans-effects of RPE on hiPSC in our recent studies. We reported that RPE secreted pigment epithelium derived-factor (PEDF) that markedly induced apoptosis in hiPSC and hESC [21]. hiPSCs or ESCs in culture inserts ceased to survive when co-cultured with RPEs. Further addition of hPEDF induced apoptosis in hiPSCs or ESCs drastically. In fact, when hiPSCs were transplanted into the subretinal space of nude rats, the log_10_TPD_50_ value was 4.73 (n = 20), whereas the value was only 2.12 (n = 30) when hiPSCs alone were transplanted subcutaneously into NOG mice with Matrigel. The 400-fold difference in the TPD_50_ values under these conditions are at least partly explained by an environmental effect related to the subretinal space, besides the difference in the status of immunodeficiency in these species or use of Matrigel. We suggest that the environmental effects of the subretinal space are mediated by PEDF secreted by RPE. The close protein sequence identity between human PEDF and the rat counterpart support this idea.

As many as 1×10^4^ hiPSC cells were required to form tumors in the subretinal space of nude rats. Similar numbers (0.8–1.5×10^4^) of hiPSC-derived RPE were transplanted into the subretinal space in the tumorigenicity test. Note that tumorigenicity testing of iPSC-derived RPE via clinical administration route will always give “negative” results, if we aim to detect a tumor from remaining small number of undifferentiated hiPSCs in final product. In this context, tumorigenicity tests conducted by transplanting serial dilutions of hiPSCs combined with hiPSC-derived RPEs into the subretinal space might not be informative. We suggest that tumorigenicity testing via clinical administration route might be useful to detect tumors from intermediate or incompletely differentiated RPE cells, but not for those relatively rare remaining undifferentiated iPSC. For these reasons, we conducted high dose (1×10^6^) subcutaneous RPE transplantation in parallel to examine tumor-forming events from rare hiPSC in hiPSC-derived RPE and full dose hiPSC-derived RPE subretinal transplantation without diluting them with hiPSCs. This was the basis for our rationale in designing multiple tumorigenicity tests for iPSC-derived RPE. As the FDA commentary report^11^ stated, the design of tumorigenicity tests should be tailored for each specific product. We hope our approach will facilitate a further discussion related to tumorigenicity testing of iPSC-derived cell products.

Considering the number of rodents used, the duration of the monitoring period, the sensitivity to detect tumors in immuno-deficient rodents via both subcutaneous and subretinal administration routes and the overall incidence of tumor formation from iPSC-derived RPE final cell products in these rodents, we conclude that the tumorigenic potential of the hiPSC-derived RPE cells produced by our methods is negligible. Of course, in considering the overall safety of the procedure in humans, discussion should include the site of transplantation as well as the source of the cells (autologous or allogeneic) and the immune-suppression status of the patients.

## Materials and Methods

All the experiments using human samples and animal studies were reviewed and approved by the IRB of the Foundation for Biomedical Research and Innovation (FBRI) and Riken Center for Developmental Biology (Riken CDB), and the committee for animal experiments of the FBRI.

### Cell Culture

The human iPSC (hiPSC) line 201B7 [12] established from dermal fibroblast with retroviruses pMXs-*POU5F1, -Sox2, -c-Myc,* and *-Klf4* (Riken Bio Resource Center, Tsukuba, Japan) was maintained on feeder layers (SNL [13]) in ReproFF2 (ReproCELL) and 5 ng/mL bFGF (Peprotech). Cell line 836B1 (supplied by CiRA Kyoto University) was established from dermal fibroblast of a healthy donor, and 59, K11, K21, 101, RNT9 or RNT10 lines were derived from dermal fibroblast of 6 patients with retinitis pigmentosa (with a photoreceptor-specific gene mutation) after obtaining informed consent from the patients. These fibroblasts were reprogrammed with episomal EBNA vectors carrying integrated *POU5F1, SOX2, KLF4, MYCL, LIN28A* and *GLIS1* (59-G, K21-G, 101-G, RNT9 and RNT10) or *POU5F1, SOX2, KLF4, MYCL, LIN28A* and *p53shRNA* (101-EV, K11-EV and K21-EV). They were established on autologous fibroblast-derived feeders and were maintained in primate ES medium (ReproCELL) with 5 ng/mL bFGF (Wako) [14]. iPSCs were differentiated into retinal pigment epithelium (RPE) as reported previously [15]. iPSC-derived RPE cell clones (59-G3 RPE, K21-G18 RPE, 101-G25 RPE, RNT9 RPE, RNT10 RPE, 101-EV RPE, K11-EV9 RPE or K21-EV15 RPE) were differentiated from the following parental iPSC clones: 59-G3, K21-G18, 101-G25, RNT9-2-8, RNT10-24, 101-EV3, K11-EV9 or K21-EV15, respectively. They were maintained in RPE maintenance medium [5],[15] [DMEM:F12 (7∶ 3) (Sigma-Aldrich) containing B-27 supplement (Invitrogen), 2 mM L-glutamine (Sigma), 0.5 nM SB431542 (Sigma-Aldrich) and 10 ng/mL bFGF (Wako)]. Human primary RPE (Lonza) was maintained in Retinal Pigment Epithelial Cell Basal Medium (Lonza Biologics, Basel, Switzerland) containing supplements [L-glutamine, GA-1000, and bFGF (Lonza)]. For transplant studies, hiPSC-derived RPE cells in suspension were collected for subcutaneous transplantation or seeded on collagen gel (collagen gel culture kit, Nitta Gelatin) to make a collagen-lined RPE monolayer or double layer cell sheet. The RPE cell sheet was maintained in F10 culture medium (Sigma) and 10% FBS for 4 weeks and RPE maintenance medium for 3 weeks and detached from the collagen gel with collagenase-1 (Roche). The RPE cell sheet was then pipetted and mixed with Matrigel for subcutaneous transplantation or dissected with laser micro dissection (LMD, Carl Zeiss) just before retinal transplantation into animals.

### Animal Studies

#### Mouse subcutaneous transplantation

Various doses of HeLa cells either embedded in 200 µL of Matrigel™ (BD Biosciences) or suspended in 200 µL of PBS (without Matrigel) were injected into subcutaneous tissue of 7- to 8-week-old female nude mice (BALB/cA, JCl-nu/nu; Clea Japan, Inc. Tokyo), SCID mice (C.B-17/Icr-scid/scid, Jcl; Clea), NOD-SCID mice (NOD/ShiJic-scid, Jcl; Clea) or NOG mice (NOD/ShiJic-scid, IL-2R γOD/S KO Jic; Clea) using a 1 mL syringe (TERMO) with a 26 G needle. Animals were monitored for 36 weeks. At the end of the experiments, mice were sacrificed and tumors were removed and fixed with 4% PFA. Paraffin sections were stained with hematoxylin and eosin (HE) for histological observation. Various doses of hiPSC 201B7 cells or 1×10^6^ hiPSC-derived RPE cells were embedded in 200 µL of Matrigel™ (BD Bioscience) or suspended in 200 µL of PBS (without Matrigel) and injected subcutaneously into 7- to 8-week-old female NOG mice using a 1 mL syringe (TERMO) with a 26 G needle and monitored for 6–15 months. At the end of the experiments, mice were sacrificed and all the transplants including RPE embedded in 200 µL Matrigel were removed with tweezers and fixed with 4% paraformaldehyde (PFA).

#### Rat subretinal transplantation

Three-week-old female nude rats (F344/NJcl-rnu/rnu; Clea) were anesthetized by intraperitoneal administration of a mixture of ketamine 100 mg/kg: xylazine 10 mg/kg (Daichi-Sankyo). The pupil of the right eye was dilated with mydriatics (0.5% tropicamide and 0.5% phenylephrine hydrochloride, Santen Pharma). A small incision was made at the right eye corner of the sclera with a 27 G needle. Then, various doses of HeLa cells, hiPSCs or 1 mm×1 mm hiPSC-RPE cell sheets in 2 µL DMEM/F12 medium were injected (Hamilton syringe with 33 G needle) into the subretinal space through the previously made incision in the sclera. The cells or the RPE sheet was transplanted just above the subretinal capillary plexus by observing the position of the Hamilton syringe needle through the dilated pupil under a surgical microscope. The subretinal capillary plexus was readily observed in albino nude rats and was used as a landmark of the subretinal space. The transplanted nude rats were monitored for 8–82 weeks. At the end of the experiments, rats were sacrificed and transplanted whole eye balls were removed and fixed with 4% PFA.

### RT-PCR and qRT-PCR

Total RNA was isolated with the RNeasy plus Mini Kit (Qiagen) in accordance with the manufacturer’s instructions. Contaminating genomic DNA was removed using a gDNA Eliminator spin column. cDNA was generated from 50 ng of total RNA using PrimeScript RT Master Mix (Takara Bio) and PrimeSTAR MAX DNA Polymerase (TaKaRa Bio). Real-time PCR was then performed with an ABI 7000 Sequence Detection System (Applied-Biosystems) and SYBR-green in accordance with the manufacturer’s instruction. Gene expression levels were normalized to that of *GAPDH*. qRT-PCR was performed using the QuantiTect Probe one-step RT-PCR Kit (Qiagen). The expression levels of target genes were normalized to those of the RNase P transcript, which were quantified using TaqMan human RNase P control reagents (Applied Biosystems). All qRT-PCR reactions were run for 45 cycles. The sequences of primers and probes used in the present study are listed in Table S1.

### *Alu* PCR

*Alu* sequences specific to human cells were used to design the primers. The *Alu* primer 5′-AAGTCGCGGCCGCTTGCAGTGAGCCGAGAT-3′ and 50 ng of DNA template, PrimeSTAR Max DNA Polymerase (Takara) were used for PCR reactions (28 cycles). The DNA templates in various ratios (human HeLa DNA: mouse NIH3T3 DNA) were used to determine the human cell detection sensitivity by *Alu* PCR. PCR products were separated by electrophoresis (MyRun, Cosmobio) with 1% agarose gel (Nacalai), and the image was digitally captured (Bio-Pyramid, Mecan).

### Immunohistochemistry

Transplanted tissues were fixed with 4% paraformaldehyde. Paraffin embedded tissue sections were stained with haematoxylin/eosin. Then, the paraffin sections were deparaffinized with xylene and sequential 100%, 95%, 80%, 70% ethanol treatments for 5 min each. The sections were treated with 10 mM citric acid (pH 6) at 95°C for 50 min followed by permeation with 0.4% Triton-X in PBS at room temperature for 30 min. The deparaffinized sections were stained with antibodies against human Lamin-A (1∶200; ab108595; Abcam), BEST1 (1∶200; ab2182; Abcam) and Ki-67 (1∶400; #9449; Cell Signaling). Nuclei were stained with Hoechst 33258 (Dojindo) and DAPI (Dojindo). hiPSC-derived RPE cells were collected in suspension and fixed with 4% paraformaldehyde followed by staining with antibodies against POU5F1 (OCT3/4) (1∶ 100; sc-5279; Santa Cruz), or BEST1 (1∶ 200; ab2182; Abcam). Antibodies were visualized with Alexa Fluor 488 goat anti-mouse (1∶ 1,000; Invitrogen) or Alexa Flour 488 goat anti-rabbit (1∶ 1,000; Invitrogen). Fluorescent microscopic images were captured with a fluorescent microscope (Olympus BX51, IX71, Tokyo, Japan).

## Conclusion

We tested the tumorigenic potential of hiPSC-derived RPE using immuno-deficient rodents. These preclinical tests laid the foundation for upcoming clinical studies using autologous hiPSC-derived RPE sheets for treatment of wet type age-related macular degeneration (AMD). One million hiPSC-derived RPE cells were transplanted subcutaneously into 65 NOG mice and 0.8–1.5×10^4^ hiPSC-derived RPE cells were transplanted into the subretinal space of 26 nude rats. No tumors were found after 6–15 months of monitoring. Considering the number of rodents used, the duration of the monitoring period, the sensitivity to detect tumors in immuno-deficient rodents, we conclude that the tumorigenic potential of the hiPSC-derived RPE cells prepared by our method is negligible.

## Supporting Information

Table S1 **Primers for RT-PCR and Alu PCR, Probes and Primers for qRT-PCR are listed.**
(DOCX)
