# Supplementary material for: Tumorigenicity Studies of Induced Pluripotent Stem Cell (iPSC)-Derived Retinal Pigment Epithelium (RPE) for the Treatment of Age-Related Macular Degeneration
Source: PLoS One. 2014 Jan 14;9(1):e85336. doi: 10.1371/journal.pone.0085336 (PMC3891869; doi:10.1371/journal.pone.0085336)
Supplement: Table S1 — Primers for RT-PCR and Alu PCR, Probes and Primers for qRT-PCR are listed. (DOCX) [file pone.0085336.s001.docx]

| Primers for RT-PCR |  |  |  |
| --- | --- | --- | --- |
| Gene | Forward primer sequence (5'→3') | Reverse primer sequence (5'→3') |  |
| *LIN28A* | CACGGTGCGGGCATCTG | CCTTCCATGTGCAGCTTACTC |  |
| *POU5F1* | GAAACCCACACTGCAGCAGA | TCGCTTGCCCTTCTGGCG |  |
| *BEST1* | ATCAGAGGCCAGGCTACTACAG | TCCACAGTTTTCCTCCTCACTT |  |
| *CRALBP* | GACTGGGGTTAAATCTCACAGC | TGACATGTTGCCTATGGAAGAC |  |
| *PAX6* | TTAACACACTTGAGCCATCACC | AAATCTCGGATGTCTGTCCACT |  |
| *TYR* | AGCCCAGCATCATTCTTCTC | GGCGTTCCATTGCATAAAGA |  |
| *GAPDH* | CGATGCTGGCGCTGAGTAC | CCACCACTGACACGTTGGC |  |
| Probes and primers for qRT-PCR |  |  |  |
| Gene | Probe sequence (5'→3') | Forward primer sequence (5'→3') | Reverse primer sequence (5'→3') |
| *LIN28A* | CGCATGGGGTTCGGCTTCCTGTCC | CACGGTGCGGGCATCTG | CCTTCCATGTGCAGCTTACTC |
| *POU5F1* | CGGACCACATCCTTCTCGAGCCCAAGC | GAAACCCACACTGCAGCAGA | TCGCTTGCCCTTCTGGCG |
| Primer for Alu PCR |  |  |  |
|  | AAGTCGCGGCCGCTTGCAGTGAGCCGAGAT |  |  |
